# Ultrasensitive single-genome sequencing: accurate, targeted, next generation sequencing of HIV-1 RNA

**DOI:** 10.1186/s12977-016-0321-6

**Published:** 2016-12-20

**Authors:** Valerie F. Boltz, Jason Rausch, Wei Shao, Junko Hattori, Brian Luke, Frank Maldarelli, John W. Mellors, Mary F. Kearney, John M. Coffin

**Affiliations:** 1 HIV Dynamics and Replication Program, CCR, National Cancer Institute, NIH, Translational Research Unit, 105 Boyles Street, Building 535 Room 111, Frederick, MD 21702-1201 USA; 2 Frederick National Laboratory for Cancer Research, Advanced Biomedical Computing Center, Leidos Biomedical Research, Inc, Frederick, MD USA; 3 Division of Infectious Disease, University of Pittsburgh, Pittsburgh, PA USA; 4 Department of Molecular Biology and Microbiology, Tufts University, Boston, MA USA

**Keywords:** HIV, Single-genome sequencing, SGS, Primer ID, Supermajority correction, Allele linkage, Targeted next-generation sequencing, NGS, Deep sequencing, Minority variants, HIV drug resistance

## Abstract

**Background:**

Although next generation sequencing (NGS) offers the potential for studying virus populations in unprecedented depth, PCR error, amplification bias and recombination during library construction have limited its use to population sequencing and measurements of unlinked allele frequencies. Here we report a method, termed ultrasensitive Single-Genome Sequencing (uSGS), for NGS library construction and analysis that eliminates PCR errors and recombinants, and generates single-genome sequences of the same quality as the “gold-standard” of HIV-1 single-genome sequencing assay but with more than 100-fold greater depth.

**Results:**

Primer ID tagged cDNA was synthesized from mixtures of cloned BH10 wild-type and mutant HIV-1 transcripts containing ten drug resistance mutations. First, the resultant cDNA was divided and NGS libraries were generated in parallel using two methods: uSGS and a method applying long PCR primers to attach the NGS adaptors (LP-PCR-1). Second, cDNA was divided and NGS libraries were generated in parallel comparing 3 methods: uSGS and 2 methods adapted from more recent reports using variations of the long PCR primers to attach the adaptors (LP-PCR-2 and LP-PCR-3). Consistently, the uSGS method amplified a greater proportion of cDNAs, averaging 30% compared to 13% for LP-PCR-1, 21% for LP-PCR-2 and 14% for LP-PCR-3. Most importantly, when the uSGS sequences were binned according to their primer IDs, 94% of the bins did not contain PCR recombinant sequences versus only 55, 75 and 65% for LP-PCR-1, 2 and 3, respectively. Finally, when uSGS was applied to plasma samples from HIV-1 infected donors, both frequent and rare variants were detected in each sample and neighbor-joining trees revealed clusters of genomes driven by the linkage of these mutations, showing the lack of PCR recombinants in the datasets.

**Conclusions:**

The uSGS assay can be used for accurate detection of rare variants and for identifying linkage of rare alleles associated with HIV-1 drug resistance. In addition, the method allows accurate in-depth analyses of the complex genetic relationships of viral populations in vivo.

## Background

Next generation sequencing (NGS) has the potential to become a powerful tool for studying the genetics of viral RNA populations from cDNA libraries generated by RT-PCR. To date, its application to targeted sequencing of diverse HIV-1 or other viral RNA populations has been limited by PCR error, unequal amplification of sequences (PCR bias), and PCR recombination during library construction. It is known that polymerase mis-incorporation errors accumulate during PCR [1–4] requiring the use of sophisticated statistical algorithms of variable accuracy [5–7] to distinguish errors from actual genetic polymorphisms. PCR bias occurs by selective or preferential amplification of some templates over others, limiting and misrepresenting the number of original distinct viral RNA templates represented in the final sequencing data [1–4]. Most importantly, PCR-mediated recombination can rearrange the sequences among amplicons producing variants or haplotypes that are not actually present in vivo [2, 8, 9], and causing the loss of rare haplotypes through recombination between amplicons with wildtype alleles that dominant the PCR reaction. As such, PCR recombination not only skews population frequencies, but also limits the ability to detect linkage among rare genetic polymorphisms. Together, these problems have restricted the applicability of NGS for genetic analysis of HIV-1 or other viral populations causing it to fall short of replacing the “gold standard” of HIV-1 single-genome sequencing (SGS), which virtually eliminates PCR error, bias, and recombination but is constrained by the limited number of sequences that can be obtained easily [10–14].

To help address these issues, primer IDs, consisting of molecular tags comprising 4–10 degenerate nucleotides (nt), have been incorporated into RT primers so that each cDNA molecule generated by reverse transcription is uniquely labeled [1, 15–18]. Primer ID-tagged cDNAs are then replicated by PCR and daughter amplicons are sequenced by NGS. Next, binning sequence reads by their common primer IDs reveals PCR template resampling. In addition, the alignment of binned sequences facilitates the identification of PCR errors and PCR recombination such that one consensus sequence can be generated that is devoid of such artifacts. Although mis-incorporation errors within the primer ID itself during PCR can give misleading results, filtering techniques can be used to detect and exclude primer IDs with mis-incorporations [19]. As such, primer IDs are extremely effective in identifying errors introduced during NGS library generation and are the only means by which the number of amplified templates can be accurately determined; and, consequently, allele frequencies in HIV-1 RNA populations accurately measured.

During library generation, NGS requires the attachment of adaptor sequences for library capture, local amplification, and sequencing. Current methods to attach these adaptors employ PCR primers of varied length, ranging from 55 [19, 20] to as long as 92 nucleotides [21]. In addition, two [19] or three rounds of flanking PCR [20] are used to attached the requisite adaptors. One such “long primer PCR” method (LP-PCR-1) has been shown to produce very high levels of PCR recombination [21] such that the final results are unreliable for identifying rare mutations or for performing accurate analyses of population genetics. Newer methods for long primer PCR (LP-PCR-2 and LP-PCR-3) [19, 20] are improvements over LP-PCR 1, but as shown here, these methods can still cause loss of variants either due to PCR bias, error, and/or recombination. Accordingly, we developed a new method for NGS library construction called ultrasensitive single-genome sequencing (uSGS), which uses a unique method to attach the NGS adaptors to amplified templates that does not require additional PCR. The method combines limited-cycle PCR with a highly efficient method of adapter ligation. The uSGS method also amplifies a higher fraction of cDNA molecules than the LP-PCR methods, and significantly reduces the percentage of sequences that are lost from PCR errors and recombination. Data resulting from uSGS rivals the accuracy and reliability of the “gold standard” HIV-1 SGS assay (sometimes referred to as single genome amplification [SGA] and sequencing) but with more than 100-fold greater sequencing depth.

Here, we report a comparative analysis of uSGS and methods adapted from recent reports [2, 19–21] for targeted NGS library generation and show that uSGS is a superior method for generating NGS datasets free of PCR error, and recombination. Although uSGS is described here for its utility in studying HIV-1 RNA populations, it can be easily modified to investigate other viral RNA populations as well as transcript sequences across cell types, including malignant cells.

## Experimental design

**Fig. 1:**
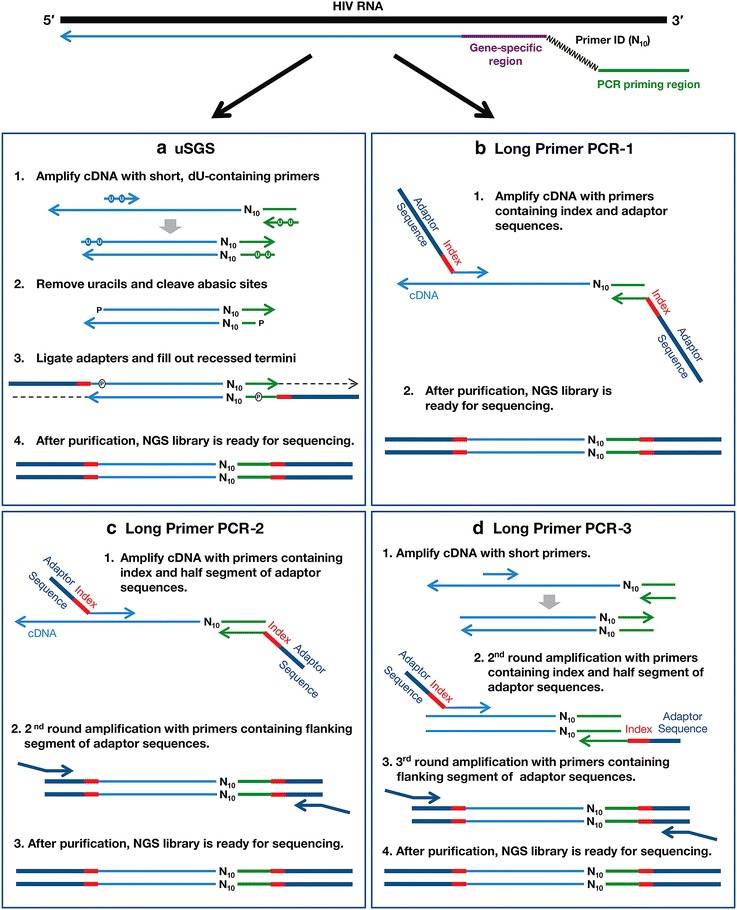
Schematic representation of the methods used for NGS library construction. A cDNA library labeled with Primer IDs (*top*) is divided and used for each method. **a** uSGS. Short PCR primers (25 and 31 bases) containing 5′ dU in place of dT residues (*dots* in the primers) are used to amplify the cDNA. Products are cleaved at the dU sites creating dsDNA with 17-nt 3′-overhangs at both ends. The ends are then ligated to the essential NGS adapters and filled out using Klenow Fragment DNA polymerase to generate a fully double-stranded NGS library. **b** Long primer PCR-1. Long primers (90 and 93nt) containing NGS adapter sequences are used to amplify the cDNA library. **c** Long primer PCR-2. Relatively long primers (50–61nt) are used in 2 rounds of flanking PCR to amplify the cDNA and attach the adaptors. **d** Long primer PCR-3 involves 3 rounds of PCR. The cDNA is amplified with short primers (25 and 31 bases) followed by 2 rounds of flanking PCR using long primers (50–61nt) to attach the adaptors

Primer ID-tagged cDNAs were prepared from mixtures of wild-type (WT) and mutant transcripts derived from cloned BH10 HIV *pol* DNA. The mutant transcripts contained 10 well-characterized, HIV-1 drug resistance mutations rendering them useful for measuring recombination and allele frequencies. In our initial experiments, the cDNA was divided and amplified with 2 different methods, uSGS (Fig. 1a) and LP-PCR-1 (Fig. 1b). uSGS was performed with short (25-31nt) primers containing 4 or 5 deoxyuridine (dU) residues. The amplified products were subsequently treated with uracil DNA glycosylase (UDG) and alkali, leaving ~17nt single-stranded tails, permitting efficient directional ligation [22, 23] of Illumina adaptor sequences. LP-PCR 1 used long (93nt) primers to incorporate Illumina adaptor sequences in a single round of PCR. In subsequent experiments, the primer ID-tagged cDNAs mixtures were divided 3-ways and amplified by uSGS (Fig. 1a) or by LP-PCR-2 (Fig. 1c) and LP-PCR-3 (Fig. 1d), the latter two methods requiring 55–65nt primers to incorporate Illumina adapters. LP-PCR-2 and 3 were performed using the reagents and conditions specified in the original publications [19, 20]. LP-PCR-2 required 2 rounds of PCR and LP-PCR-3 required 3 rounds. NGS libraries prepared by all methods were sequenced using paired-end MiSeq technology and raw sequencing reads were processed through the analytical pipeline described in Methods. Briefly, sequence reads from samples were separated according to their indexes and binned by common primer IDs. “Super-consensus” sequences were built from “qualified” unique primer ID groups resulting in a single-genome sequence per primer ID. To be qualified, a “super-consensus” sequence required two characteristics. First, it had to be derived from a set of common primer IDs that satisfied the cutoff model designed by Zhou et al. [19]. Zhou et al. [19] showed that low abundance primer IDs or “offspring” were artifacts resulting from PCR errors within the primer ID. To ensure that these artifacts were eliminated from the data, a cutoff model was designed for the minimum number of raw sequence reads required to make a consensus sequence. Second, we required “supermajorities” of ≥80% identity at each nucleotide position for a “super-consensus” sequence to be included in the dataset. We found that a simple majority (i.e., >50% consensus at each nucleotide position) does not eliminate artifacts resulting from early-cycle PCR recombination or other PCR errors. In a simple majority, only late cycle PCR errors or late cycle recombinant events are eliminated. Accordingly, we increased the stringency of our filtering so that a “supermajority” of ≥80% identical bases at each nucleotide position would eliminate early PCR cycle errors and recombinants. To illustrate our findings, we analyzed the mixtures of WT and mutant transcripts at majority cutoffs of 50–100% for the 10 HIV-1 drug resistance sites for each of the methods. Accordingly, if a final consensus sequence had a site of ambiguity, the sequence was discarded because this sequence contained either a PCR mis-incorporation error or a mixture of calls at the same site due to PCR recombination (or rare use of the same primer ID more than once). Final datasets for all the approaches were analyzed to compare (1) the fraction of the total cDNA molecules amplified efficiently, (2) the frequency of PCR/sequencing errors, (3) the level of PCR-based recombination, and (4) the sensitivity for detection of rare alleles.

## Results

### PCR efficiency, and template sampling

First, we compared amplification efficiency resulting from 3 independent experiments in which a single preparation of cDNA was synthesized using primer IDs, divided and used to compare the LP-PCR-1 and uSGS methods for NGS library generation. Table 1 tallies the average number of unique primer IDs containing the qualified number of raw sequences above the cutoff as defined by Zhou et al. [19]. The % cDNA amplified was calculated by dividing the number of consensus sequences above the cutoff by the number of starting copies as measured by qPCR, with averaging over 3 experiments. As shown in Table 1A, uSGS yielded more total unique primer IDs and more than twice as many consensus sequences compared to LP-PCR-1 (30 vs. 13% of starting cDNAs, respectively). To further assess which method of library construction provided more complete sampling of the viral population, libraries were synthesized in 3 more replicate experiments in which a preparation of primer ID-tagged cDNA was divided into thirds and used to compare LP-PCR-2, LP-PCR-3, and uSGS methods (Table 1B). Ultrasensitive SGS generated greater than two-fold more consensus sequences than LP-PCR-3 (31 vs. 14%) and 1.5-fold more sequences than LP-PCR-2 (31 vs. 21%).

**Table 1:** Comparison of cDNA amplification efficiency among methods using the same HIV-1 site specific RT-Primer ID primer

	uSGS		LP-PCR-1	
	AVG	STDV	AVG	STDV
A.				
Copies of cDNA by qPCR	134,193	45,940	134,193	45,940
Total # unique primer IDs^a^	39,597	7491	17,614	6442
% cDNA amplified	30%	13%	13%	3.3%

	uSGS		LP-PCR-2		LP-PCR-3	
	AVG	STDV	AVG	STDV	AVG	STDV
B.						
Copies of cDNA by qPCR	81,040	21,192	81,040	21,192	81,040	21,192
Total # unique primer IDs^a^	25,111	9046	15,794	1392	11,565	482
% cDNA amplified	31%	4.9%	21%	2.5%	14%	2.2%

All results are taken from an average of 3 separate experimental libraries prepared from each method

^a^Total number of consensus sequences above the Zhou algorithm cutoff [19]

### PCR and sequencing error rates

Binning raw sequence reads by primer ID, generating alignments, and producing “supermajority” sequences (≥80% agreement at each site) resulted in PCR/sequencing error rates approximately 10-fold lower than the uncorrected sequences regardless of the method used for generation of NGS libraries. Specifically, uncorrected PCR/sequencing error rates of 2.8 × 10^−3^ and 1.6 × 10^−3^ were calculated for raw sequence reads generated by LP-PCR-1,2,3 and uSGS, respectively, values consistent with other reports [2, 24]. After applying the Zhou et al. [19] consensus cutoff model and generating the supermajority sequences from the alignments of reads sharing common primer IDs, the PCR/sequencing error rates were reduced to 1 × 10^−4^ for uSGS, LP-PCR-1 and LP-PCR-3 and to 2 × 10^−4^ for LP-PCR-2 (Table 2). These rates are comparable to those measured for standard SGS and the basal rate of 1 × 10^−4^ mutations per base per cycle reported for HIV-1 reverse transcriptase in vitro [25], indicating that PCR and sequencing errors were essentially eliminated by the inclusion of primer IDs and the generation of supermajority sequences by our pipeline. Furthermore, in a separate experiment using 100% WT transcripts, we found that the PCR/sequencing error rate was approximately the same—5 × 10^−5^—at all sites in the 500 base pair amplicons indicating that PCR/sequencing errors do not occur at higher rates at the drug resistance sites and that no false positive calls for drug resistance mutations occurred.

**Table 2:** Comparison of Recombination between methods at different consensus majority cutoffs in mixtures of BH10 WT and mutant transcript RNA

% Majority cutoff	Method/enzyme	Total sequences	% Sequences excluded^a^	% Sequences remaining	% Remaining recombinants missed	% Error^b^
50	uSGS	33,870	0.6	99.4	0.39	0.020
60		33,870	1.1	98.9	0.30	0.018
70	Kapa Hi Fi	33,870	2.6	97.4	0.20	0.015
80	Uracil+	33,870	*5.8*	*94.2*	*0.13*	*0.014*
90		33,870	28.4	71.6	0.12	0.010
100		33,870	43.9	56.1	0.12	0.009
50	LP-PCR-1	11,008	4.7	95.3	4.07	0.033
60		11,008	11.7	88.3	1.86	0.030
70	Taq Gold	11,008	27.3	72.7	0.34	0.020
80		11,008	*45.0*	*55.0*	*0.20*	*0.014*
90		11,008	77.3	22.7	<0.01	0.007
100		11,008	87.2	12.8	<0.06	0.006
50	LP-PCR-2	23,142	1.4	98.6	0.91	0.037
60		23,142	3.3	96.7	0.62	0.033
70	Kapa2G	23,142	10.5	89.5	0.30	0.024
80	Robust	23,142	*25.4*	*74.6*	*0.14*	*0.020*
90		23,142	63.5	36.5	0.09	0.013
100		23,142	85.7	14.3	0.15	0.009
50	LP-PCR-3	20,252	4.6	95.4	6.44	0.016
60		20,252	13.8	86.2	2.00	0.015
70	Platinum	20,252	23.4	76.6	0.35	0.013
80	Hi Fi Taq	20,252	*34.8*	*65.3*	*0.15*	*0.011*
90		20,252	71.7	28.3	<0.04	0.004
100		20,252	92.0	8.0	<0.07	0.004

^a^Consensus sequences were excluded due to failure to achieve the required majority at each level of consensus at each nucleotide position, likely due to in vitro PCR recombination

^b^Incorrect bases at non drug resistant sites

### PCR-mediated recombination

Given that low frequency alleles can be missed and linkage eliminated by PCR recombination, we assessed its extent using all 4 methodologies. Primer ID tagged cDNAs were generated from mixtures of mutant and WT BH10 HIV-1 *pol* transcripts and NGS libraries were prepared. NGS libraries were sequenced, reads binned by primer ID, consensus cutoffs of >50, ≥60, ≥70, ≥80, ≥90 and 100% majorities applied, and consensus sequences were generated from the survivors (Table 2) at each of the 10 drug resistance sites. Where a mixture was detected at any position in a specific primer ID bin at less than the stated majority, that consensus sequence was eliminated from consideration. The final data set was then analyzed for recombinants that were missed by the filtering pipeline at the ten drug resistance sites and for incorrect bases at all non-drug resistant sites. As italicized in Table 2, uSGS at the ≥80% supermajority achieved the most accurate data set resulting in an error rate equivalent to that of RT in vitro [25], while preserving 94.4% of the original cDNA sequences. By contrast, the other 3 methods were able to achieve the same accuracy at the ≥80% supermajority cutoff but with a much greater cost to the number of final sequences, with reductions in the size of the final data set ranging from 25 to 45%. Because recombination only destroys linkage of alleles, detecting linkage among drug resistance sites would be hampered in libraries generated from clinical samples using LP-PCR-1, 2 or 3 given the in vitro recombination rates observed, whereas detection of such linked alleles would be much more likely in libraries generated using uSGS.

**Fig. 2:**
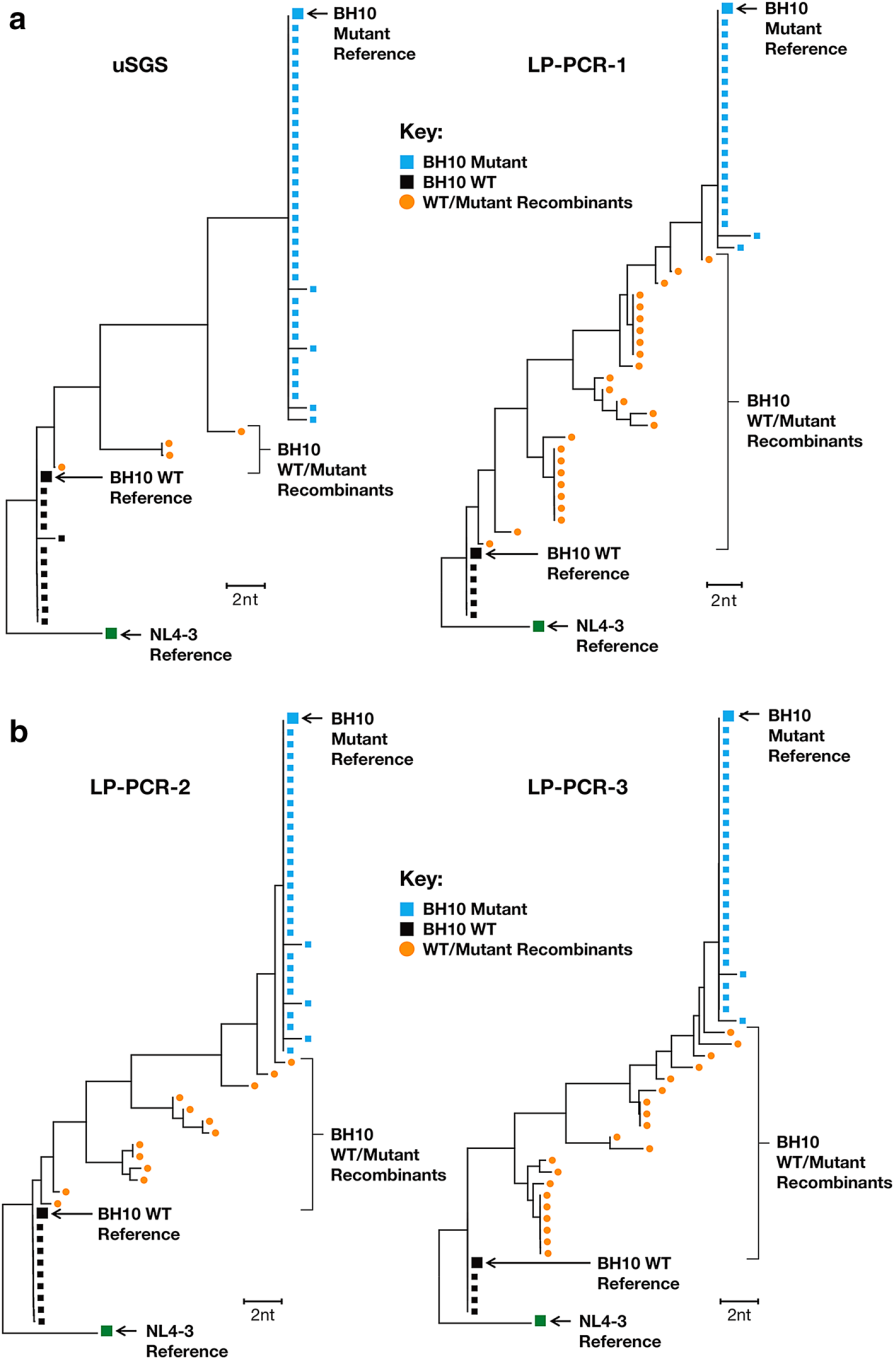
Neighbor joining trees comparing PCR recombination in each method. Neighbor joining trees rooted on NL4-3 generated from randomly selected sets of 50 supermajority sequences obtained from mixtures of WT and Mutant BH10 *pol* transcripts. Reference sequences for the BH10 mutant and WT and NL4-3 WT are shown in large *blue*, *black* and *green squares*, respectively. Sequences matching the references are shown in the *same colors* as the references. The *orange circles* show an intermediate step in the bioinformatics computations and represent those sequences identified as PCR recombinant species that would be lost from the respective data sets after they were deleted in the final steps of the pipeline

To further illustrate this finding, neighbor-joining (NJ) trees were generated for a random selection of 50 supermajority consensus sequences produced from the mixtures sequenced using each method (Fig. 2). Also shown on the trees are the BH10 WT and mutant HIV-1 reference sequences. The orange circles represent sequences with mixtures of nucleotides at one or more of the 10 ten drug resistance sites, which failed to satisfy the supermajority requirement and therefore would be deleted from the dataset. In this random sampling, 50, 26 and 40% of the sequences would have been be omitted from the LP-PCR-1, LP-PCR-2 and LP-PCR-3 datasets respectively compared to only 8% from the uSGS dataset.

### Calculating allele frequencies

To compare the ability of all methods to accurately determine allele frequencies, cDNA libraries were obtained from mixtures of mutant RNA transcripts in a WT background from 10 to 1% for uSGS vs. LP-PCR-1 (Table 3a, b) and from mixtures of 30–0.3% for uSGS vs. LP-PCR-2 and LP-PCR-3 (Table 3d, e). Each cDNA mixture was divided in half for the first comparison described above and in thirds for the second. To determine whether PCR recombination results in the loss of sensitivity for detection of individual alleles, consensus sequences where built from the >50 and ≥80% supermajority of reads. Recombinants were retained for this analysis (Table 3). An obvious loss of mutant alleles toward the 5′ end of the transcripts was evident in the LP-PCR-1, 2, and 3 but not in the uSGS data (Table 3a–e). This loss of mutant alleles, most evident in the ≥80% majority cutoff (Table 3b, e), was likely due to premature DNA polymerase (or RT) termination followed by extension of the termination product on the more abundant WT template during the early cycles of PCR in the LP-PCR-1,2,3 protocols. Consistent with this hypothesis, the allele frequencies in the raw sequence data from these methods (without generating consensus sequences) did not exhibit such bias (Table 3c) due to the fact that the individual alleles were still present regardless of whether they were in recombinant species or not.

**Table 3:** Comparison of individual allele frequencies from different mixtures of BH10 WT and Mutant RNA transcripts analyzed by all methods

Method	qPCR cDNA input copies	Consensus sequences	Allele frequency expected (%)	% allele frequency detected									
				65R	67N	70R	74V	100I	103N	181C	184V	188C	190A
a. uSGS vs Long Primer PCR-1 at 50% majority cutoff													
uSGS	179,825	41,050	10	15.2	15.7	15.7	15.7	15.8	15.8	16.0	16.0	16.0	16.0
LP-PCR-1	179,825	32,492	10	1.90	2.00	2.03	1.07	2.80	2.94	7.04	7.06	7.07	7.08
uSGS	178,175	28,061	1	1.53	1.60	1.58	1.59	1.62	1.62	1.65	1.65	1.65	1.64
LP-PCR-1	178,175	20,565	1	0.22	0.23	0.24	0.12	0.31	0.32	0.69	0.70	0.70	0.70
b. uSGS vs Long Primer PCR-1 at 80% majority cutoff													
uSGS	179,825	41,050	10	8.2	8.5	8.5	8.5	8.5	8.5	8.5	8.5	8.5	8.5
LP-PCR-1	179,825	32,492	10	0.07	0.07	0.07	0.07	0.07	0.07	0.27	0.27	0.27	0.27
uSGS	178,175	28,061	1	0.39	0.40	0.39	0.40	0.40	0.40	0.40	0.40	0.41	0.41
LP-PCR-1	178,175	20,565	1	0.02	0.02	0.02	0.02	0.02	0.02	0.04	0.05	0.04	0.04
c. uSGS vs Long Primer PCR-1 *without Primer ID consensus builds*													
uSGS	179,825	41,050	10	18	18.8	18.9	18.8	18.5	18.75	19	19.5	19.5	19.4
LP-PCR-1	179,825	32,492	10	6.8	8.5	8.4	6.9	8.6	8.8	9.2	9.3	9.0	8.8
uSGS	178,175	28,061	1	1.8	2.0	1.9	1.9	1.9	1.9	2.1	2.1	2.1	2.1
LP-PCR-1	178,175	20,565	1	0.83	0.74	0.83	0.58	0.78	0.8	0.96	1.3	0.86	0.86
d. uSGS vs Long Primer PCR-2 and Long Primer PCR-3 at 50% majority cutoff													
uSGS	69,000	23,048	30	30	31	32	31	32	32	32	32	32	32
LP-PCR-2	69,000	17,230	30	21	22	22	22	22	23	23	23	23	23
LP-PCR-3	69,000	14,229	30	23	24	24	24	25	26	28	28	28	28
uSGS	74,800	17,270	3	4.7	4.9	4.9	4.9	4.9	4.9	4.9	4.9	4.9	4.9
LP-PCR-2	74,800	14,451	3	3.1	3.2	3.3	3.3	3.3	3.3	3.3	3.3	3.3	3.3
LP-PCR-3	74,800	10,915	3	0.6	0.7	0.7	0.8	2.2	2.6	4.2	4.2	4.2	4.2
uSGS	67,860	16,757	0.3	0.35	0.38	0.36	0.36	0.36	0.37	0.37	0.37	0.36	0.36
LP-PCR-2	67,860	15,696	0.3	0.32	0.30	0.33	0.31	0.32	0.36	0.32	0.32	0.32	0.32
LP-PCR-3	67,860	9546	0.3	0.02	0.02	0.02	0.03	0.02	0.10	0.31	0.31	0.32	0.32
e. uSGS vs Long Primer PCR-2 and Long Primer PCR-3 at 80% majority cutoff													
uSGS	69,000	23,048	30	29	30	30	30	30	30	30	31	31	31
LP-PCR-2	69,000	17,230	30	12	12	12	13	17	17	21	22	22	22
LP-PCR-3	69,000	14,229	30	1.8	1.9	2.1	2.1	4.4	5.3	24	25	26	26
uSGS	74,800	17,270	3	4.3	4.5	4.5	4.5	4.6	4.6	4.6	4.9	4.9	4.9
LP-PCR-2	74,800	14,451	3	1.1	1.1	1.2	1.2	2.0	2.0	3.2	3.3	3.3	3.3
LP-PCR-3	74,800	10,915	3	<0.01	<0.01	0.01	0.01	0.02	0.02	2.6	3.0	3.2	3.2
uSGS	67,860	16,757	0.3	0.28	0.31	0.29	0.29	0.32	0.32	0.33	0.36	0.36	0.36
LP-PCR-2	67,860	15,696	0.3	0.11	0.10	0.10	0.11	0.21	0.22	0.30	0.32	0.31	0.32
LP-PCR-3	67,860	9546	0.3	<0.01	<0.01	<0.01	<0.01	<0.01	<0.01	0.12	0.12	0.17	0.19

### Usage of different enzymes

To determine if the DNA polymerase used was the basis for the superior performance of uSGS compared to the other 3 methods, we conducted three parallel experiments in which uSGS or LP-PCR-1 were used to generate NGS libraries from mixtures of WT and mutant RNAs while varying the DNA polymerase used. AmpliTaq Gold (used in LP-PCR-1), Kapa Uracil + DNA polymerase (used for uSGS) and Platinum Taq (used in LP-PCR-3) were compared (Table 4). For all comparisons, the uSGS method, regardless of the enzyme used, provided a more complete sampling of the viral population, maintaining more of the starting cDNA represented in the final data set, as well as, retaining ≥90% of the final sequences after PCR errors and recombinants were removed (Table 4). While the Kapa Uracil +enzyme is an important component of the uSGS reaction because of its ability to copy dU-containing templates, we suggest that the use of shorter primers and efficient ligation are more important for optimizing the performance of the method. Moreover, it cannot be presumed that using a polymerase with the highest reported fidelity would reduce the error rate in any PCR method because polymerase processivity (or lack thereof), not fidelity, is the enzymatic property most likely to contribute to PCR recombination.

**Table 4:** Comparison of different DNA polymerases for library preparation using LP-PCR-1 and uSGS

Method	Enzyme	cDNA starting copy	Qualified sequences	% Sequences excluded^a^	Final # sequences	% Population represented
LP-PCR-1	AmpliTaq Gold^b^	34,094	11,215	40	6729	20
uSGS	AmpliTaq Gold^b^	34,094	11,859	7	11,060	32
LP-PCR-1	Kapa Uracil+^b^	34,094	3566	11	3188	9
uSGS	Kapa Uracil+^b^	34,094	18,855	2	18,512	54
LP-PCR-1	Platinum Taq^c^	14,410	2789	82	558	4
uSGS	Platinum Taq^c^	88,560	25,773	10	22,938	26

^a^Final number of “super majority” consensus sequences after removal of >2 ambiguous sites likely due to in vitro PCR recombination

^b^Synthesis of cDNA from WT/Mutant mixture of transcript HIV-1 RNA, divided into 4 parts and parallel libraries sequenced

^c^Synthesis of cDNA from WT/Mutant mixtures of transcript HIV-1 RNA independently in two separate experiments

### Comparison of uSGS to LP-PCR for analysis of a clinical sample

**Fig. 3:**
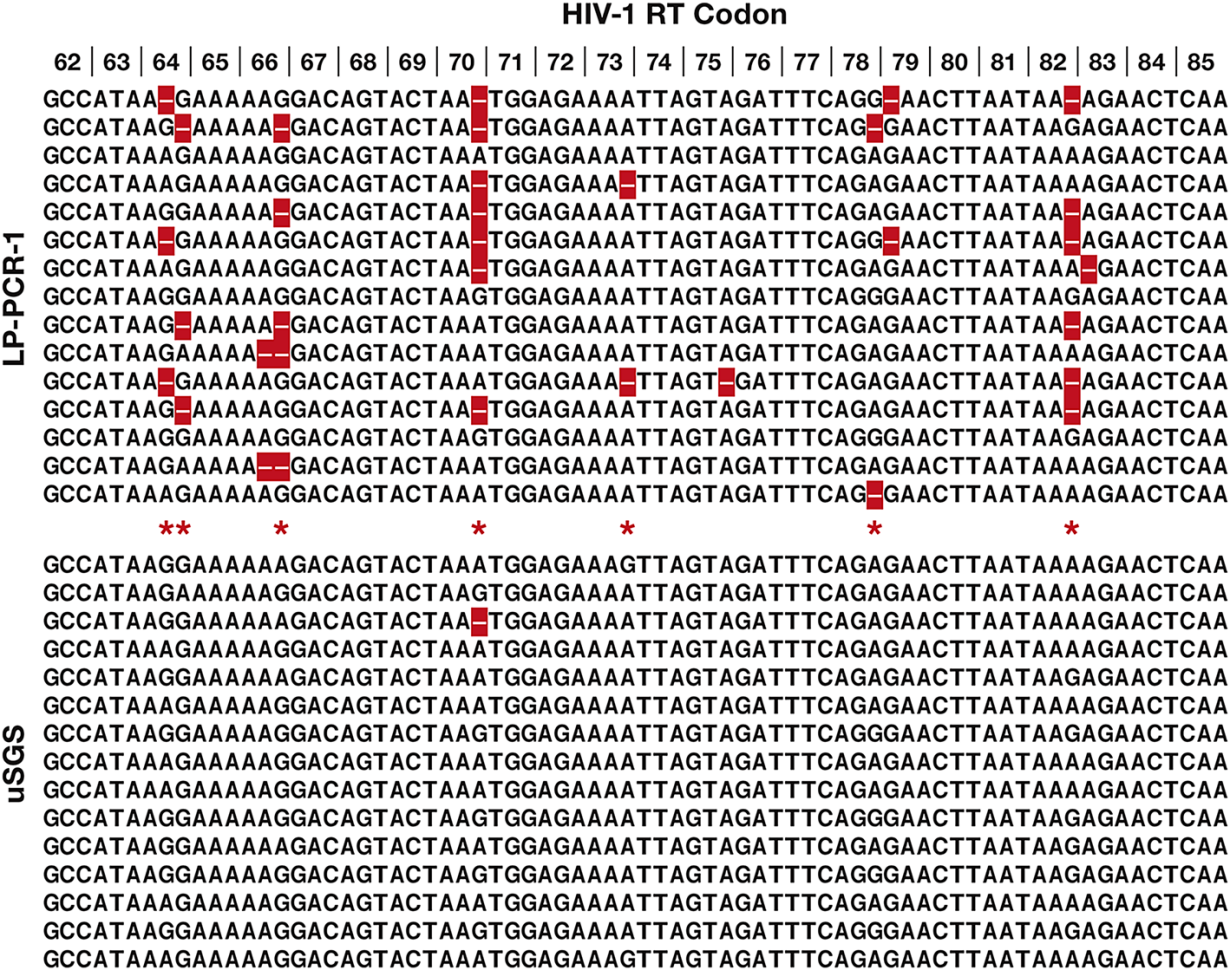
Snapshot of sequence alignments of library construction obtained from clinical sample. Small subsets of supermajority sequence alignments obtained from a donor sample using the (*a*) LP-PCR or (*b*) uSGS methods of NGS library construction. *Dashes* (“–” in *red*) which have been placed in the consensus sequences by the bioinformatics pipeline in positions with <80% identity in sequences in a given daughter set, which is indicative of recombination during PCR. *Asterisks* mark positions with diverse bases in the uSGS data where no PCR recombination is seen

To compare the methods for clinical samples, NGS libraries were constructed using LP-PCR-1 and uSGS on primer ID-containing cDNA prepared from a plasma sample collected from an untreated, chronically HIV-1 infected donor. The product (~13,600 copies of cDNA) was then divided in half for parallel library construction using LP-PCR-1 or uSGS. LP-PCR and uSGS recovered 2091 and 2237 supermajority consensus sequences, respectively. Random subsets of 15 aligned supermajority sequences were selected to assess the extent of in vitro recombination (Fig. 3). Only 3 of the 15 supermajority sequences analyzed from the LP-PCR-1 method were found to accurately reflect their parent cDNA, with the 12 remaining sequences containing ambiguous base calls (shown as dashes) among the 72 nt positions represented in the figure. The ambiguous sites are in areas of higher diversity in the sequence, suggesting that the sequences were PCR recombinants. By contrast, the uSGS method produced only a single ambiguous base indicating rare PCR recombination and the suitability of this approach for analyzing linkage and population structure after identifying and omitting such recombinants. After the filtering pipeline was applied to discard consensus sequences with >2 ambiguous bases, uSGS preserved 96% of the original cDNA sequences whereas the LP-PCR-1 dataset preserved <40%.

### Application of uSGS to clinical samples

**Fig. 4:**
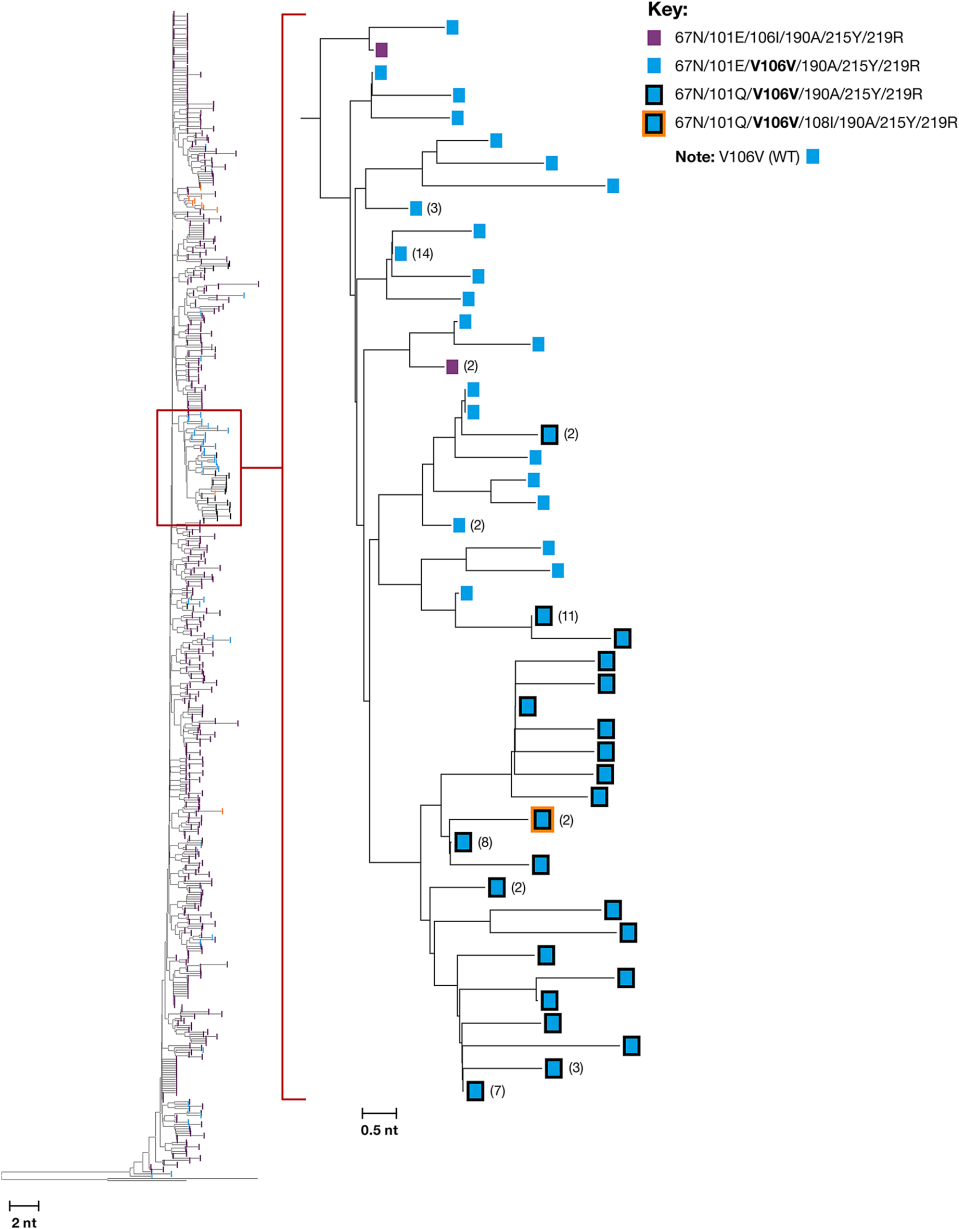
Analysis of HIV-1 population structure in a clinical sample using uSGS for library construction. Neighbor joining trees of HIV-1 *pol* sequence from donor 1 sample 1 and blow up of NJ subtree, showing clustering and linkage of WT at RT position 106 (V106V *blue squares*) with 101Q (*blue square* with *black outline*). *Numbers* of identical sequences are shown in *parentheses* as this NJ tree was extracted from 1585 unique SGS, where identical sequences were collapsed. Within the highlighted subtree, note especially two sequences (2) in which WT RT codon 106, and rare mutations 101Q and 108I (*blue square* with *black* and *orange out line*) were found to be linked. Detection of such a rare linkage event would be virtually impossible using LP-PCR or conventional SGS. Data were obtained by NGS uSGS and rooted on Consensus B

To assess the robustness and sensitivity of the uSGS assay, we applied the assay using 1 ml from each of 3 plasma samples with viral loads of 5000–10,000 copies/ml from two HIV-infected donors on failing antiretroviral therapy. uSGS data were assessed for unique linkage among HIV-1 drug resistant variants and for population structure using neighbor-joining (NJ) trees (Table 6; Fig. 4), which allow visualization of the alignments without imposing any evolutionary assumptions on the relationships between sequences.

Of the 3470 total consensus sequences from all 3 samples, an average of 1% were lost to analysis due to >2 ambiguous bases from in vitro recombination, resulting in 677–1577 final sequences per sample, or 30–80 fold more sequences than are customary for standard SGS (usually 20–50 sequences). The NJ tree of the sequences from donor 1 (Fig. 4) revealed clusters of resistant variants on multiple independent nodes. Within these clusters, rare HIV-1 variants (haplotypes) were detected in each sample. For example, in the sample from patient 1 (sample 1), the frequencies of RT codons 106 V (WT), 101Q and 108I (mutants) were approximately 9, 4 and 0.5%, respectively (Table 5). If one assumes that these frequencies are mutually independent, the expected frequency of the haplotype containing all three alleles is ~0.002% (Table 6). However, we found that the frequency of the 101Q/106V/108I haplotype was 0.13% (Table 6 and expanded section of NJ tree in Fig. 4), significantly different (p = 0.0004) from the expected frequency. In addition, 4 of the 5 haplotypes containing 106 V in the cluster were present at significantly higher frequencies than expected from mutual independence (p = <1.0 × 10^−237^ to 0.004; Table 6). These results imply that the clustering of alleles observed in the neighbor joining analyses (Fig. 4) could not have arisen by chance reshuffling during PCR and therefore accurately represent the HIV-1 population in vivo.

**Table 5:** Frequency of resistance mutations in donors failing anti-retroviral therapy as measured by uSGS

Allele	Donor 1 Sample 1 % Frequency (1585 uSGS)	Donor 1 Sample 2 % Frequency (675 uSGS)	Donor 2 Sample 3 % Frequency 1227 (uSGS)
D67N	100	99.85	99.92
T69A	<0.06	0.15	0.08
T69I	0.06	<0.15	<0.08
K70N	<0.06	<0.15	0.08
K70Q	<0.06	<0.15	1.07
K70T	<0.06	<0.15	1.23
L74I	<0.06	<0.15	0.08
K101E	95.56	95.72	<0.08
K101Q	4.12	3.99	0.08
K101R	<0.06	<0.15	0.49
V106I	91.12	91.58	0.33
V108A	<0.06	<0.15	0.08
V108I	0.51	<0.15	0.41
M184I	<0.06	<0.15	0.16
M184V	<0.06	<0.15	99.84
Y188C	0.06	<0.15	<0.08
G190A	99.81	100	<0.08
G190E	<0.06	<0.15	0.08
G190R	<0.06	<0.15	0.08
G190T	*0.13*	<0.15	<0.08
T215F	0.06	<0.15	<0.08
T215R	<0.06	<0.15	<0.08
T215Y	99.94	100	100
K219R	99.94	99.85	<0.08

**Table 6:** Linkage of resistance mutations in donors failing anti-retroviral therapy as measured by uSGS

Patient/sample	Haplotypes Mutant codons in italics	Expected (%)	Observed (%)	P value
1/1	*67N*, 69T, *101E*, *106I, 108V,* 188Y*, 190A, 215F, 219R*	0.05	0.06	0.58
	*67N,* 69T*, 101Q,* 106V*, 108I,* 188Y*, 190A, 215Y, 219R*	0.002	0.13	0.0004
	*67N,* 69T*, 101E,*106V,108V,188Y,*190A,215Y,219R*	8.41	5.58	1.18*10^−5^
	*67N,*69T*,101E,*106V*,108I,*188Y,*190A,215Y,219R*	0.04	0.06	0.49
	*67N,*69T,*101Q,*106V,108V,188Y*,190A,215Y,219R*	0.36	3.04	1.0*10^−237^
	*67N,*69T,*101Q,*106V,108V,188Y,190G*,215Y,219R*	0.0002	0.06	0.004
	*67N,* 69T, *101E, 106I, 108I,* 188Y, *190A, 215Y, 219R*	0.44	0.25	0.18
	*67N,* 69T, *101E, 106I,* 108V, 188Y, *190T, 215Y, 219R*	0.11	0.13	0.52
	*67N,* 69T, *101E, 106I,* 108V, 188Y, *190A,215Y, 219R*	86.25	89.22	0.0003
	*67N, 69T, 101E, 106I, 108V, 188Y, 190A, 215Y, 219K*	0.05	0.06	0.58
	*67N, 69T, 101K, 106I, 108V, 188Y, 190A, 215Y, 219R*	0.34	0.38	0.46
	*67N, 69T, 101Q, 106I, 108V, 188Y, 190A, 215Y, 219R*	3.72	0.82	3.1*10^−13^
	*67N, 69I, 101E, 106I, 108V, 188Y, 190A, 215Y, 219R*	0.05	0.06	0.58
	*67N, 69T, 101E, 106I, 108V, 188C, 190A, 215Y, 219R*	0.05	0.06	0.58
	*67N, 69T, 101Q, 106I, 108I, 188Y, 190A, 215Y, 219R*	0.02	0.06	0.26
1/2	*67N,* 69T, *101E, 106I, 190A, 215Y,* 219K	0.13	0.15	0.58
	*67N,* 69T, 101K, 106V, *190A, 215Y, 219R*	0.02	0.15	0.15
	67D, 69T, *101E,* 106V, *190A, 215Y, 219R*	0.01	0.15	0.08
	*67N,* 69T, *101E, 106I, 190A, 215Y, 219R*	87.42	90.25	0.01
	*67N,* 69T, *101Q,* 106V*, 190A, 215Y, 219R*	0.33	3.10	5.1*10^−14^
	*67N,* 69T, 101K, *106I, 190A, 215Y, 219R*	0.27	0.15	0.45
	*67N,* 69T, *101E,* 106V, *190A, 215Y, 219R*	8.04	5.02	0.001
	*67N, 69A, 101E, 106I, 190A, 215Y, 219R*	0.13	0.15	0.58
	*67N,* 69T*, 101Q, 106I, 190A, 215Y, 219R*	3.64	0.89	6.1*10^−6^
2/3	*67N,* 69T*, 70Q,* 74L, 101K, 106V, 108V, *184V,* 190G, *215Y*	1.05	1.07	0.51
	*67N,* 69T, 70K, 74L, *101R,* 106V, 108V, *184V,* 190G, *215Y*	0.48	0.49	0.52
	*67N,* 69T, 70K, 74L, 101K, 106V, 108V, *184I,* 190G, *215Y*	0.16	0.16	0.57
	67D, 69T, 70K, 74L, 101K, 106V, 108V, *184V,* 190G, *215Y*	0.08	0.08	0.62
	*67N.*69T, 70K, 74L, 101K, *106I,* 108V, *184V,* 190G, *215Y*	0.32	0.33	0.54
	*67N,* 69T, 70K, 74L, 101K, 106V, 108V, *184V, 190E, 215Y*	0.08	0.08	0.62
	*67N* 69T, 70K, 74L, 101K, 106V, 108V, *184V, 190R, 215Y*	0.08	0.08	0.62
	*67N,* 69T, 70K, *74I*, 101K, 106V, 108V, *184V*, 190G, *215Y*	0.08	0.08	0.62
	*67N 69A*, 70K, 74L, 101K, 106V, 108V, *184V*, 190G, *215Y*	0.08	0.08	0.62
	*67N*, 69T, *70N*, 74L, 101K, 106V, 108V, *184V*, 190G, *215Y*	0.08	0.08	0.63
	*67N* 69T, *70T*, 74L, 101K, 106V, 108V, *184V*, 190G, *215Y*	1.21	1.23	0.51
	*67N*, 69T, 70K, 74L, *101Q*, 106V, 108V*184V*, 190G, *215Y*	0.08	0.08	0.62
	*67N* 69T, 70K, 74L101K, 106V, 108V, *184V*, 190G, *215Y*	95.76	95.64	0.44
	*67N* 69T, 70K, 74L, 101K, 106V, *108A*, *184V*, 190G, *215Y*	0.08	0.08	0.62
	*67N*, 69T, 70K, 74L, 101K, 106V, *108I*, *184V*, 190G, *215Y*	0.40	0.41	0.53

## Discussion

SGS was originally developed to circumvent errors inherent in earlier methods of analysis of the genetic structure of viral populations, including PCR induced mutation and recombination, as well as resampling of the same templates. NGS is being increasingly utilized by researchers for the same purpose, and offers significant advantages over SGS in expense and throughput. However, in its simplest form this new technology is subject to the same errors—mutation, recombination, and resampling—that SGS was designed to prevent. Great care must be taken to ensure that errors introduced by the method of library construction are minimized, and are not falsely interpreted as being characteristic of the population being analyzed. Methods (e.g. Nextera [26]) that do not permit the targeted identification of individual molecules or determination of the numbers of cDNA molecules that have been synthesized, amplified, and sequenced cannot be used to accurately reconstruct intrapatient population structure or genetic relationships of viral RNA or DNA populations. As described here and in recent studies, the use of primer IDs using different methods of library preparation and analysis to track each molecule and its amplified progeny during NGS library construction has helped to resolve this problem [2, 19, 20, 27]. However, the methods used to date to generate such targeted libraries remain subject to early cycle PCR-recombination and or early cycle PCR error resulting in sequence data that may not be entirely accurate and could have the potential to miss low frequency alleles. In fact, we observed a decrease in the sensitivity of detecting some mutant alleles toward the 5′ end of the transcript using the LP-PCR-1, 2 and 3 methodologies. The striking 5′ to 3′ bias, likely reflects small amounts of premature termination products in the initial cDNA (i.e. truncated cDNAs). Since the same cDNA was used for all the analyses, the bias most likely arises from reduced priming efficiency of the extended primers used in the LP protocols relative to that of our short primers or the truncated cDNA sequences. In the first PCR cycle, the completed cDNA products will be copied into plus strands, which will in turn be copied initiating on to the added primer or the 3′ truncated cDNA. The frequency of 5′ biased recombination in this step will be a function of the relative priming efficiency of the two primers (truncated cDNA vs. reverse LP primer). We hypothesize that the non-complementary 5′ extensions (index and adaptor sequence) of the LPs greatly reduces their efficiency in the second cycle (relative to the truncated cDNAs), when only a small 3′ portion of the LP primer is paired with the 3′ end of the first cycle product, allowing recombinant products primed by the truncated cDNAs to predominate. Since the truncated cDNAs will prime randomly on the first cycle products, this effect will cause 5′–3′ biased loss of minority alleles in the final PCR products. Consistent with this concept, when allele frequencies were determined using the raw sequence data, no bias was encountered because all individual alleles were counted irrespective of recombination. However, without using primer IDs, the alleles are in a background of PCR and sequencing errors and so there is no ability to distinguish background from actual genetic polymorphism or to infer the correct population structure due to resampling artifacts. These results highlight not only that detection of linkage among rare variants is hampered in LP-PCR-1, 2 or 3 methods from in vitro recombination in the early cycles of PCR, but that the uSGS method can correct this deficiency. For this reason, we developed a method of library preparation for targeted NGS of HIV-1 RNA, which results in very low early cycle PCR-recombination and more complete sampling of cDNA libraries. Combined with a more stringent bioinformatics pipeline for filtering out early PCR errors, this approach results in datasets that are virtually free of PCR error and PCR recombination. Consequently, our uSGS methodology is the most effective means for studying HIV-1 population structure as well as for detecting linkage among rare alleles. Critical features of this methodology include (1) amplification of primer ID-tagged cDNA molecules using short primers and a more robust DNA polymerase that catalyzes PCR uniformly and efficiently [28] and (2) creation of 17 base 3′ overhangs at both ends of the amplicons to promote highly efficient ligation of linkers containing NGS adaptors [22, 23], which eliminates the need for a subsequent PCR amplification step. Other protocols requiring that NGS adapters be attached to dsDNA libraries by blunt-end or nearly blunt-end ligation, (i.e., via single nucleotide 3′ overhangs) or in vitro transposition (Nextera^®^) are relatively inefficient [29]. Only small fractions of amplicons are successfully appended in these reactions, necessitating subsequent rounds of PCR prior to sequencing [3, 29], likely resulting in amplification bias.

As previously reported, mutation arising in primer IDs during PCR amplification have the potential to generate artifacts during NGS library production [15–19]. To address this issue, we have also included in our analysis pipeline methods developed by Zhou et al. [19] that identify and exclude such false primer IDs. These tools allow us to employ primer IDs without being misled by artifacts produced during library amplification.

In conclusion, the uSGS method for targeted, massively parallel sequencing of HIV-1 RNA-derived cDNA libraries (1) results in a higher fraction of cDNAs being amplified and sequenced and (2) virtually eliminates in vitro recombination and PCR error, resulting in hundreds to thousands of single-genome HIV-1 sequences that accurately reflect the population genetics of the parent RNA. This method can also be used to determine linkage of low frequency HIV-1 drug resistance mutations and can be modified to assess RNA populations of other viruses or expressed host genes.

## Methods

### RNA preparation and cDNA synthesis

Mixtures of WT and multidrug resistant mutant HIV-1 BH10 *pol* RNA transcripts were derived from cloned viral DNA as previously described by Shao et al. [2]. An 895-bp region of HIV-1 BH10 WT and mutant transcripts containing codons 22–291 in HIV-1 *pol* was used for controls. Mixtures of WT and multidrug resistant transcripts containing 10 well characterized drug resistance mutations, distributed along the 546nt fragment, were used for analyzing in vitro recombination. Viral RNA from donor plasma was extracted as described previously and placed on ice [30]. RNAs were reverse transcribed in 50 μL reactions that included 30 nM final concentration of HIV-1 gene specific primer with Primer ID (GSPID 2834R), 500 μM dNTPs (Promega C1145), 1× First Strand Buffer, 1 mm DTT, 20 U RNase out (Promega Cat #N2115; 40 U/µl) and 200 U SuperScript III (Life Technologies, Cat #18080-044; 200 U/µl). Oligonucleotide sequences are provided in the “Appendix”.

Reverse transcription reactions were incubated at 45 °C for 1 h and quenched to 4 °C. 60 units of Exonuclease 1 (NEB: Cat #M0293S), and 1U Shrimp Alkaline Phosphatase (NEB: Cat #M0371S) were added and incubated as follows: 37 °C for 30 min and 85 °C for 15 min. 5 units of RNase H (NEB: Cat #M0297S) were added and incubated for 20 min at 37 and 85 °C for 15 min. The cDNA was precipitated overnight at −20 °C with 0.1 volume (v) 3 M Sodum Acetate, pH 5.5, 20 µg glycogen (Roche: Cat #1090139300) and 3v 95% ETOH. The washed and dried precipitated cDNA was suspended in 5 mM Tris–HCl, pH 8.0 and processed reserving 5 µl for quantification by qPCR.

### cDNA amplification in uSGS

Five replicate 25 µl PCR reactions were performed for the first round of PCR with final concentrations as follows: 1× Kapa Hi Fi Hot Start Uracil +reaction mix (KAPABIOSYTEMS Cat #KK2802), 300 nM forward primer #28F (2195), 300 nM dU-containing reverse primer PrimRegion-R-5Us and cycled as follows: 1 cycle 95 °C for 3 min, 10 cycles 95 °C for 20 s, 60 °C for 30 s, 68  for 2 min with 5 s added onto each subsequent cycle. The products from all 5 reactions belonging to the same sample were combined and purified using the Mini Elute PCR purification kit from Qiagen (Cat #28004). A second round of PCR was performed exactly as the first except the entire purified product from the first PCR was used as template in 5 replicate reactions with final concentrations of 300 nM primers of forward primer 2286-F-dUs and reverse primer PrimRegion-R-5Us and cycled 20–30 times. The products from all 5 reactions belonging to the same sample are combined and PCR purified using the QIAquick PCR Purification Kit from Qiagen (Cat #28104).

### Processing of DNA termini and adapter ligation in uSGS

In 50 μl reactions, PCR products generated using dU-containing primers were treated with 10 units of uracil-DNA glycosylase (UDG) (NEB M0280S) and incubated for 2 h at 37 °C. This enzyme hydrolyzes the glycosidic bonds in 2′-deoxyuridines, thereby releasing the uracil bases and generating chemically labile abasic sites at the affected positions. The UDG-treated DNA was cleaved at these abasic sites with 2 N NaOH at a final concentration of 0.25 N and incubated at 65 °C for 30 min. Solutions were neutralized by adding an equimolar amount of 2 M Trizma Hydrochloride. DNAs were renatured by heating to 85 °C for 2 min, and slow cooled at 0.1 °C/s to 25 °C. The dsDNA was precipitated overnight at −20 °C with 0.1v 3 M Sodium Acetate, pH 5.5, 20 µg glycogen and 3v 95% ETOH. Collectively, these treatments generated a double-stranded DNA library in which amplicons contained distinct 17 nt 3′ overhangs at both ends. These sticky ends were complementary to the NGS adapters (Linker F1 and Linker R2), facilitating their attachment to the hydrolyzed DNA strands by bridging ligation – a process much more efficient than blunt end ligation or ligation utilizing a single nucleotide overhang, avoiding inefficient ligation and the necessity for additional cycles of PCR.

The method was completed by hybridizing NGS linkers to the 17-nt 3′ overhangs using ~2 pmol DNA, 1× NEB buffer #2, 4 pmol linker F, 4 pmol linker R and 1 mM final concentration of ATP (NEB Cat #P0756S). The reactions were heated to 65 °C for 5 min and slow cooled at 0.1 °C/s to 25 °C. 400 units of T4 DNA ligase (NEB Cat #M0202S) was added and incubated at 25 °C for 16 h (overnight), and then heated to 65 °C for 10 min. The 5′ overhangs were filled in with 5 units of Klenow Fragment DNA polymerase (NEB Cat #M0212S) and 400 µM dNTPs and incubated at 37 °C for 2 h.

Ligation reactions were resolved on a 1.5% agarose gel and the ~719 bp fragment was excised and purified using Qiagen Qiaquick Gel Extraction kit Cat #28704.

Each sample was quantified using the KAPA SYBR FAST universal qPCR kit (KK4824 KAPA Biosystems) by following manufacturer’s directions. Samples were normalized to 2 nM combined and loaded for paired-end sequencing using the Illumina Miseq.

### cDNA amplification in LP-PCR-1

Five replicate 25 µl PCR reactions were performed for LP-PCR-1 with final concentrations of 1× SYBR green buffer, 4 mM MgCl_2_, 500 µM dNTPs, 5U AmpliTaq Gold and 300 nM each MiSeq forward primer I and MiSeq reverse primer 2. All reactions were performed using the following cycling conditions: 1 cycle 95 °C for 10 min, 10 cycles 95 °C for 30 s, 53 °C for 30 s, 72  for 2 min, then 45 cycles of 95 °C for 30 s, 72 °C 2 min. Following PCR, the 5 reactions were combined and gel purified using a Qiaquick gel extraction kit as per manufacturer’s protocol. Samples were quantified following manufacturer’s protocol using a Kapa Library Quantification kit.

### cDNA amplification in LP-PCR-2

This protocol was adapted from Zhou et al. [19] and was essentially 2 rounds of 25 cycle flanking PCR using oligos of 50 and 54 nt in length for the first round and 58 and 61 nt for the flanking round 2. In short, 5 replicate 25 µl PCR reactions were performed for LP-PCR-2, round 1 with final concentrations of 1× Robust KapaG2 mix (Kapa Biosystems) and 200 nM forward primer B2F and reverse B6R and cycled as follows: 1 cycle of 95° 1 min, 25 cycles of 95 °C 15 s, 58 °C1 min and 72 °C 30 s and 1 cycle of 72 °C for 3 min. PCR products were PCR purified using the QIAquick PCR Purification Kit from Qiagen and 2 µl (of the 50 µl eluted) used as template in a second round of PCR. Round 2 was performed exactly as round 1 except forward primer B6F and reverse B7R were used. All reactions were performed using the same cycling conditions as in round 1.

### cDNA amplification in LP-PCR-3

This protocol was adapted from Seifert et al. [20] and comprised 3 rounds of PCR. The first round used short oligos of 25–31 nt in length in 30 cycles of PCR, the second used 30 cycles of flanking PCR using oligos of 50 and 54 nt long and the third used 12 cycles of flanking PCR using oligos of 58 and 61 nt in length. In short, 5 replicate 25 µl PCR reactions were performed for LP-PCR-3 round 1 with final concentrations of 1× Platinum taq buffer, 400 µM dNTPs, 2 mM MgSO4, and 200 nM forward primer #28F (2195), 200 nM reverse primer PrimRegion-R (the same primers used in uSGS round 1) and cycled as follows: 95 °C 2 min, 30 cycles at 95 °C 15 s, 53 °C 30 s, 68 °C 1 min. PCR products were diluted to ~100,000 copies (as measured by qPCR) and used as template in a semi nested PCR round 2. Round 2 was performed exactly as round 1 except forward primer B1F and reverse B4R were used. A third round of PCR was performed following purification of the products and 1 ng used as template in 12 additional cycles of PCR using forward primer B6F and reverse B7R and JumpStart Taq ready mix (Sigma-Aldrich P2893) and cycled as follows: 95 °C 2 min, 12 cycles of 95 °C 30 s, 53 °C 30 s, 72 °C 1 min. The 5 reactions were combined and gel purified using a Qiaquick gel extraction kit as per manufacture’s protocol. Samples were quantified following manufacture’s protocol using Kapa Library Quantification kit.

### MiSeq sequencing and analyses

Samples were prepared for Miseq Illumina sequencing as directed in the protocol for the 500-cycle MiSeq v2 kit (MS-102-2003 Illumina Inc, San Diego, CA). A final concentration of ~10 pM of the pooled sample was spiked with 20% of the PhiX DNA control. After the Miseq runs were complete, Fastq files were exported for bioinformatics analyses. The MiSeq paired-end reads were concatenated (the reverse complement sequence was used for read 2). Low quality reads were removed using a program available at http://hannonlab.cshl.edu/fastx_toolkit (with parameters −Q20 –P90, and default for all other settings). The filtered fastq sequences were then converted to fasta format and the fasta reads were sorted by barcodes using programs available on the same website. The reads were then compared to a reference sequence and the primer IDs were determined using an in-house Perl script. Primer IDs containing indels were discarded. Reads with identical primer IDs were grouped for consensus or super-majority (≥80% identity at each site) construction. The minimum number of raw sequences for consensus construction was determined by the Zhou et al. [19] cutoff model. All primer ID groups smaller than this cutoff were discarded. For the purpose of comparing the methods presented in this manuscript, super-majority sequences containing ambiguous bases were retained. However, for analysis of clinical samples, the presence of more than one such base resulted in exclusion of that sequence from the dataset, essentially omitting the in vitro PCR recombinants from the final data. An in-house Perl script was used to determine the allele frequencies at each position. Neighbor-joining trees were constructed using Mega 6 [31]. Probabilities for the expected vs. observed haplotypes were determined with Hardy–Weinberg statistics. Miseq data will be made available in GenBank. Perl scripts used in the above analyses are available at the GitHub code repository at https://github.com/ShaoFred/MiSeq_consensus_builder.git.

## Appendix

### Reverse transcription primer all methods

GSPID-2834-R:

5′ GGTATCGAAGTCATCCTGCTAGNNNNNNNNNNTGGAGTTCATAACCCATCCAAAG 3′

### uSGS primers and linkers

PCR:

28-2195F: 5′ AAACAATGGCCATTGACAGAAGA 3′
2286-F-dUs: 5′ C***dU***GAAAA***dU***CCA***dU***ACAA***dU***ACTCCAGTATTTGC 3′
PrimRegion-R-5Us: 5′ GG***dU***A*dU*CGAAG***dU***CA***dU***CC***dU***GCTAG 3′

NGS linkers (for ligation):

Linker-F

5′ AATGATACGGCGACCACCGAGATCTACACTCTTTCCCTACACGACGCTCTTCCGATCTNNNNCGCCTGTCTGAAAATCCATACAAT 3′

Linker-R

5′ CAAGCAGAAGACGGCATACGAGATCGGTCTCGGCATTCCTGCTGAACCGCTCTTCCGATCTNNNNGCCATGTGGTATCGAAGTCATCCT 3′

### Long primer PCR-1 primers

MiSeq F1:

5′ AATGATACGGCGACCACCGAGATCTACACTCTTTCCCTACACGACGCTCTTCCGATCTNNNNCGTGATAATACTCCAGTATTTGCCATAA 3′

MiSeq R2:

5′ CAAGCAGAAGACGGCATACGAGATCGGTCTCGGCATTCCTGCTGAACCGCTCTTCCGATCTNNNNCACTGTGGTATCGAAGTCATCCTGCTAG 3′

### Long primer PCR-2 primers

First round

B2F

5′ACGACGCTCTTCCGATCTNNNNCGTGATGCCTGAAAATCCATACAATACTCCAG

B6R

5′TGAACCGCTCTTCCGATCTNNNNTAGTTGGGTATCGAAGTCATCCTGCTAG

Second round

B8F

5′AATGATACGGCGACCACCGAGATCTACACTCTTTCCCTACACGACGCTCTTCCGATCT

B7R

5′CAAGCAGAAGACGGCATACGAGATCGGTCTCGGCATTCCTGCTGAACCGCTCTTCCGATCT

### Long primer PCR-3 primers

First round

28-2195F: 5′ AAACAATGGCCATTGACAGAAGA 3′
PrimRegion-R-5Us: 5′ GGTATCGAAGTCATCCTGCTAG 3′

Second round

B1F

5′ ACGACGCTCTTCCGATCTNNNNCGCCTGGCCTGAAAATCCATACAATACTCCAG

B4R

5′ TGAACCGCTCTTCCGATCTNNNNGCCATGGGTATCGAAGTCATCCTGCTAG

Third round

B8

5′ AATGATACGGCGACCACCGAGATCTACACTCTTTCCCTACACGACGCTCTTCCGATCT

B7R

5′CAAGCAGAAGACGGCATACGAGATCGGTCTCGGCATTCCTGCTGAACCGCTCTTCCGATCT

### QPCR primers

qPCR 24F: 5′ AATACTCCAGTATTTGCCATAA 3′
qPCR 23R: 5′ TCCCCACCTCAACAGATGTT 3′

Indexes are underlined in the primer or linker sequences shown and are changed for every sample. Only examples are shown here, however, standard indexes used in MiSeq experiments are provided by Illumina, Inc. 9885 Towne Centre Drive San Diego, CA 92121 (www.illumina.com). We advise varying indexes in consecutive experiments so that cross-contamination may be readily detected.
